# In Vitro Antibacterial Experiments of Qixingjian Decoction and Its Synergistic Interaction with Oxacillin against Clinical Isolates of Methicillin-Resistant *Staphylococcus aureus*

**DOI:** 10.1155/2022/1488141

**Published:** 2022-02-16

**Authors:** Siyuan Lv, Tingxuan Huang, Ying Lin, Xingwei Yao, Huiyong Yu, Guoxing Liu, Yue Zhang, Tong Liu, Huan Liang, Chengxiang Wang

**Affiliations:** ^1^Beijing University of Chinese Medicine Third Affiliated Hospital, Beijing, China; ^2^Dongzhimen Hospital Affiliated to Beijing University of Chinese Medicine, Beijing, China

## Abstract

**Background:**

With the widespread use and abuse of antimicrobial drugs, the problem of bacterial resistance is becoming increasingly prominent. The clinical detection rate of drug-resistant bacteria is increasing year by year, so there is an urgent need to develop new antimicrobial drugs. Qixingjian Decoction (QXJT) is a formula commonly used in Chinese medicine for the treatment of sepsis caused by acute purulent infections of the face, hands, and feet. There are many compounds with antimicrobial effects that are available, but little is known about their mode of action. In this study, we mainly evaluated the antimicrobial activity of QXJT and explored its synergistic interaction with oxacillin (OX) and the mechanism of its antimicrobial activity.

**Methods:**

The antimicrobial activity of QXJT against methicillin-resistant *Staphylococcus aureus* (MRSA) was determined by the microdilution method, the broth macrodilution method, and the time-kill curve method. The main compounds in QXJT were analyzed by ultra-performance liquid chromatography. The synergistic interaction of QXJT and oxacillin (OX) was determined by checkerboard assay, and the antimicrobial mechanism of QXJT, OX, and QXJT + OX was evaluated by transmission electron microscopy (TEM) technique. The expression of MRSA superantigen virulence factors (sea, seb, and tst), and drug resistance gene (mecA) was detected to provide a new strategy for new antibiotic drugs.

**Results:**

QXJT exhibited antimicrobial activity against both clinical isolates of MRSA, MICs ranging from 18.75 to 37.5 mg/mL. Active substances such as *Scutellarein, Scutellarin, Apigenin*, and *Wogonin 7-O-glucuronide* were detected in the phytochemical analysis that may be associated with the antimicrobial activity of QXJT. The synergistic effect of QXJT and OX was determined by checkerboard assay (FICI = 0.5), and TEM images showed that QXJT could cause the disruption of MRSA cell wall, and QXJT + OX could produce greater disruption of MRSA cell wall, elucidating the synergistic effect of the two together on cell wall disruption by microscopic mechanisms. Our study shows that the combination of QXJT and OX can inhibit the expression of MRSA virulence factor, reduce the virulence of MRSA, and have no significant effect on the expression of MRSA resistance gene mecA.

**Conclusion:**

The results of this study provide scientific experimental data for the traditional application of QXJT and initially explore the mechanism of action of QXJT combined with OX.

## 1. Introduction

Since ancient times, nature plants have been used in medicine and are still used today [1]. Nature plants have been the most important source of antibiotic lead compounds and they also can be used to respond to the growing antibiotic resistance crisis [2]. The major strength of nature plants lies in their various antibacterial modes of action and the proven clinical effectiveness of plant extracts from which they are isolated [3].

Traditional Chinese medicine (TCM) has been practiced for thousands of years, and many of the herbal drugs and decoctions, particularly classical TCM decoctions, are still being used in modern Chinese medicine [4]. Qixingjian decoction (QXJT) has been clinically used in traditional Chinese medicine for more than 700 years [5, 6]. It was first recorded in 1617 in The Authentic Book of Surgery and was used for treating sepsis caused by acute infections.

*Staphylococcus aureus* has emerged as a leading etiologic agent of sepsis, owing to its propensity to cause deep-seated tissue infection and bacteremia [7]. The advent of antibiotics reduced *S. aureus* bacteremia mortality from 80% to a still unacceptable 15–50% [8]. In 1961, shortly after the introduction of methicillin for treating infections of *S. aureus*, Jevons et al. [9] identified drug-resistant *S. aureus* strained in the United Kingdom and named them MRSA.

Since 1990, the widespread of MRSA in hospitals and communities and the increasing resistance to commonly used antibiotics have made MRSA a severe threat to public health worldwide [10]. Thus, novel antimicrobials and/or new approaches to combat these problems are urgently needed [11]. The use of combination therapy can broaden the spectrum of antimicrobial activity, minimize the emergence of resistant microbial variants, and sometimes result in synergistic interaction, thereby exhibiting antimicrobial activity greater than would be expected from each antimicrobial drug individually [7]. We made a lot of efforts in the preexperiment, combining QXJT with more than 10 antibiotics with different mechanisms of action, and found that QXJT and OX was the most ideal combination. This study also tried to explore the synergistic mechanism of QXJT + OX.

The diversity and severity of MRSA diseases are partly due to the pathogen's ability to regulate the expression of multiple virulence factors [12]. The development of antivirulence strategies that interfere with bacterial toxins or virulence factors is currently attracting the attention of many researchers in various fields [13]. This study explored this perspective and may provide new strategies for exploring new antibiotic drugs.

## 2. Materials and Methods

### 2.1. Drugs

**Figure 1:**
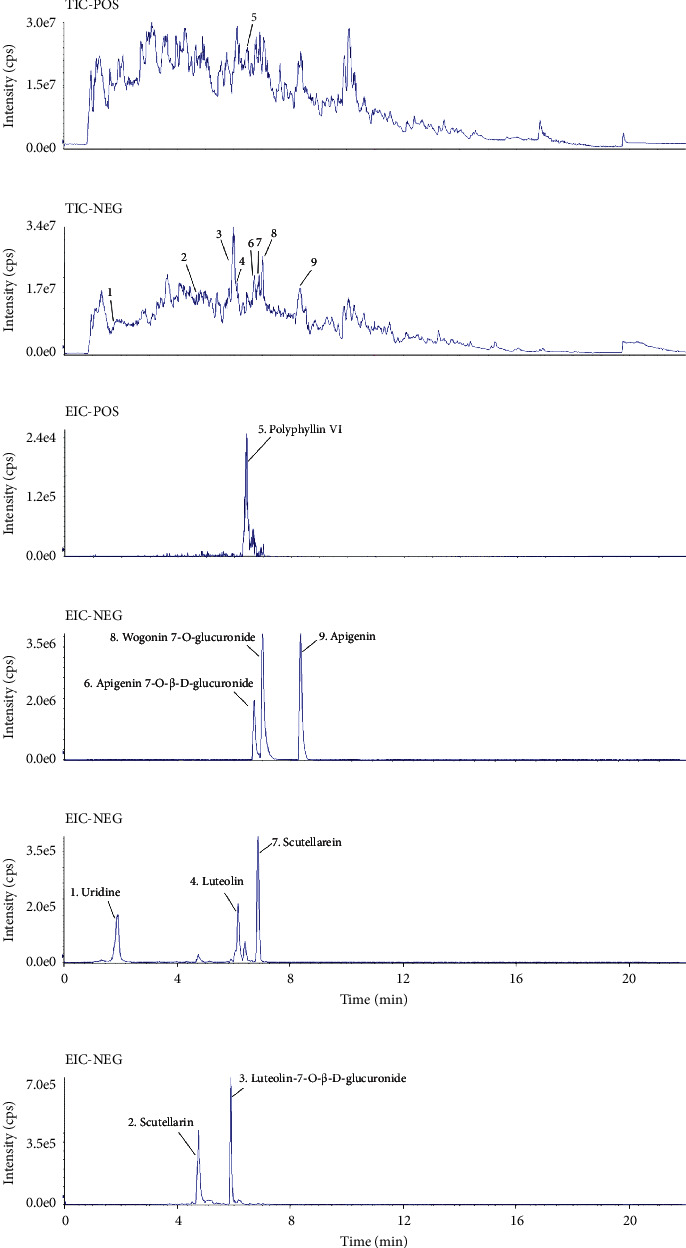
Total ion chromatography (TIC) on positive (a) and negative (b) and extraction ion chromatography (EIC, (c)–(f)).

QXJT was purchased from Beijing TRT Group (Beijing, China) and identified by Professor Wang Chengxiang of Beijing University of Chinese Medicine. QXJT consists of *Wild Chrysanthemum* (9 g), *Fructus Xanthii* (9 g), *Herba Siegesbeckiae* (9 g), *Scutellaria Barbata* (9 g), *Herba Violae* (9 g), *Ephedra* (3 g), and *Paris polyphylla* (6 g). The above herbs were refluxed and extracted with a 10-fold quantity of deionized water for 1 h in a 1 L electric heating jacket and then filtered through 8 layers of gauze. The drug residue was further refluxed and extracted with an 8-fold quantity of deionized water for 1 h and then filtered through 8 layers of gauze. The two filtrates were combined and concentrated to 300 mg/mL by rotary evaporator and stored in a refrigerator at −60°C. Before the in vitro experiment, the product was sterilized in an autoclave at 0.1 MPa and 120°C for 15 min. The quality of the herbs was consistent with the standards of Chinese Pharmacopoeia (2020). The chemical fingerprint of QXJT (ultraperformance liquid chromatography-Q Extract hybrid quadrupole orbitrap high-resolution accurate mass spectrometry [UHPLC-Q-orbitrap HRMS]) was analyzed (Figure 1) (Table 1), and the detailed method of UHPLC-Q-orbitrap HRMS is provided in the supplemental data.

Oxacillin (product name: Oxacillin sodium capsule, serial number: 210503, Sichuan Pharmaceutical Preparation Co., LTD.) was purchased from the Third Affiliated Hospital of BUCM and configured to a concentration of 64 mg/ml using phosphate-buffered saline (PBS) as a positive drug control. Before the experiment, the positive drug was used as 0.22 *μ*l membrane for bacteria removal.

### 2.2. Bacterial Strains and Culture Conditions

The tested MRSA strains (2007118 and 2008043) were kindly provided by Prof. Lin Ying isolated from clinical patients (Dongzhimen Hospital, Beijing University of Chinese Medicine, Beijing, China). All bacterial strains were cultured according to the guidelines of the Clinical and Laboratory Standards Institute (CLSI) [14].

### 2.3. Growth Curve of MRSA by Microplate Reader Measure

The growth curve of MRSA was performed according to Xu et al. [15] with some modifications. The optimum detection wavelength of MRSA absorbance was selected at 630 nm. 50 ml sterile centrifuge tubes were taken; we added 1 ml of bacterial solution (bacterial solution concentration 5 × 10^5^ CFU/mL) and incubated it for 24 h at 37°C and 200 r/min in a shaker. The bacterial growth was monitored every two hours until 24 h. The OD 630 nm value was analyzed by statistical software. Then growth curve of MRSA was plotted to determine the optimal culture time in an in vitro bacteriostatic test.

### 2.4. Evaluation of Minimum Inhibitory Concentrations (MICs)

**Figure 2:**
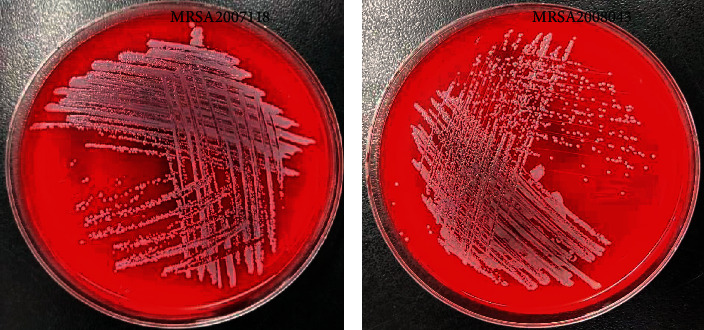
(a) MRSA2007118 image of blood plate with three streaking method. (b) MRSA2008043 image of blood plate with three streaking methods.

For the determination of MICs, the broth macrodilution method and the broth microdilution method were approved by Clinical and Laboratory Standard Institute (CLSI) with some modifications [14]. According to the preexperiment results, the wells around the 96-well plate were left empty because evaporation would affect optical density (OD) value, and the control (QXJT) group was added to exclude the effect of the excessive dark color of QXJT. QXJT was diluted with twofold serial dilutions to a final concentration of 2.34–75 mg/mL. Oxacillin (OX) was diluted with twofold serial dilutions to a final concentration of 0.125–8 mg/mL. Bacteria were collected, washed, and diluted with phosphate-buffered saline (PBS) to 5 × 10^5^ CFU/mL. MIC analysis was performed in microtitration plates with 100 *μ*l QXJT or OX, 10 *μ*l bacterial suspension, and 100 *μ*l Mueller–Hinton broth followed by incubation at 37°C for 24 h (Figure 2(a)). Each experiment was performed three times. MIC was defined as the lowest concentration, which prevented visible (naked eye) bacterial growth.

For broth macrodilution method test, QXJT was diluted with twofold serial dilutions to a final concentration of 2.34–75 mg/mL. Tubes were inoculated with the test microbes. Tubes were incubated using a shaker incubator at 37°C for 24 h.

### 2.5. A Time-Kill Assay

A time-kill assay was performed according to CLSI guidelines (15). MRSA was cultured overnight and was adjusted to a 5 × 10^5^ CFU/mL suspension with phosphate-buffered saline (PBS) (pH 7.4). Inocula were treated at 37°C with prescribed concentrations (1/4 MIC∼8 MIC) of QXJT and with normal saline as a negative control. The OD630 nm value was monitored every two hours until 24 h and was analyzed by statistical software. Each experiment was performed three times.

### 2.6. Checkerboard Assay

The checkerboard assay, commonly used for measuring interactions [16], was used to determine synergy between the OX and QXJT. The range of drug concentrations used in the checkerboard assay was the dilution range included the MIC for each drug used in the analysis. The fractional inhibitory concentration (FIC) was derived from the lowest concentrations of the OX and QXJT in combination, permitting no visible growth of the test organisms in the Mueller–Hinton broth after incubation for 24 h at 37°C. The FIC index was calculated as FICI = MIC A drug combination/MIC A drug alone + MIC B drug combination/MIC B drug alone. In antimicrobial combination, Schelz et al. [17] defined synergy as FICI ≤ 0.5, additivity as 0.5 < FICI ≤ 1, indifference as 1 < FICI ≤ 4, and antagonism as FICI > 4.

### 2.7. Transmission Electron Microscopy

MRSA was cultured overnight and was adjusted to a 5 × 10^5^ CFU/mL suspension with phosphate-buffered saline (PBS) (pH 7.4) and then was treated by 1/2 MIC QXJT, 1/2 MIC OX (preexperiment proved that it is not easy to collect a sufficient amount of Samples by the concentration above 1/2 MIC), incubated at 37°C for 24 h. To collect a sufficient amount of samples, the tubes were centrifuged at 2000 r/min for 6 minutes. Samples were rinsed two times with PBS (operated gently before fixing samples and minimized the number of rinses to avoid damage) and added 2.5% glutaraldehyde to fix at 4°C for 3 hours. Samples were rinsed again with PBS 3 times and were dehydrated with a graded acetone series and embedded in Spurr's resin. Thin slices were cut with an RMC MT-7000 ultramicrotome (Ventana) and stained with 1% uranyl acetate and Reynold's lead citrate before being seen on a transmission electron microscope at 80 kV (H-7500; Hitachi).

### 2.8. Quantitative Reverse-Transcription PCR

To compare the effect of 1/2MIC as well as MICs QXJT, OX, and QXJT + OX (derived from the checkerboard dilution method) on the expression of MRSA drug-resistant genes (mecA) as well as superantigen virulence genes (tst, sea, and seb), 1 ml of the above concentrations was added to test tubes, respectively, and 0.1 ml of MRSA bacterial solution and 1 ml of broth were added in each tube and incubated for 24 h. Quantitative reverse-transcription PCR (RT-qPCR) was performed as previously described by Li et al. [18] with some modifications. Total RNA was extracted using a bacterial RNA extraction kit for qRT-PCR according to the manufacturer's instructions. Total RNA (0.2 *μ*g) was reverse-transcribed into cDNA using EasyScript First-Strand cDNA Synthesis SuperMix (TransGene, Beijing, China). Reactions were performed in triplicate using a Power SYBR®Green PCR Master Mix (Applied Biosystems, Warrington, UK) and the Applied Biosystems 7500 system. The mRNA expression levels of the genes were normalized to the 16S rRNA gene, compared with 0.9% NaCl-treated bacterial, and quantified by the 2^−ΔΔCT^method. The sequences of the primers are presented in Table 2.

## 3. Statistical Analysis

All data were representative of the results of at least three independent experiments. For in vitro experiments, the MIC values were presented as number. Data for the growth curve of MRSA and the time-kill assay were presented as the mean ± standard deviation (S.D.). Quantitative reverse-transcription PCR randomized design with three replications was performed. Comparison between two groups with normal distribution and homogeneity of variance was analyzed using the unpaired *t*-test. A value of *p* < 0.05 was regarded as statistically significant. All data were analyzed using SPSS 25.0 software (IBM, Armonk, NY, USA) or GraphPad Prism software.

## 4. Results

### 4.1. Growth Curve of MRSA by Microplate Reader Measure

**Figure 3:**
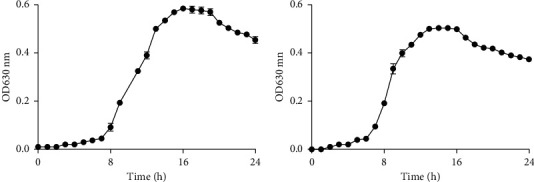
(a) Growth curve of MRSA2007118 (OD630 nm values) at different culture time points. (b) Growth curve of MRSA2008043 (OD630 nm values) at different culture time points.

After isolation and purification, the clinical strains were identified as MRSA (Figure 2) by Biomeere's VITEKAMS30 identification system after culture. The growth curves of MRSA strains were plotted in three independent experiments. As shown in Figure 3, within 24 h, OD 630 nm value and MRSA concentration gradually increased as the culturing time increased. MRSA2007118 and MRSA2008043 had little difference. It could be preliminarily judged that the growth rule of MRSA is th following: 0∼10 h slow period, 10∼14 h logarithmic growth period, 15∼18 h stability period (plateau), and >18 h decline period. According to the growth curve of MRSA, the optimal culture time of in vitro bacteriostatic test was determined to be 18∼24 h.

### 4.2. Evaluation of Minimum Inhibitory Concentrations (MICs)

**Figure 4:**
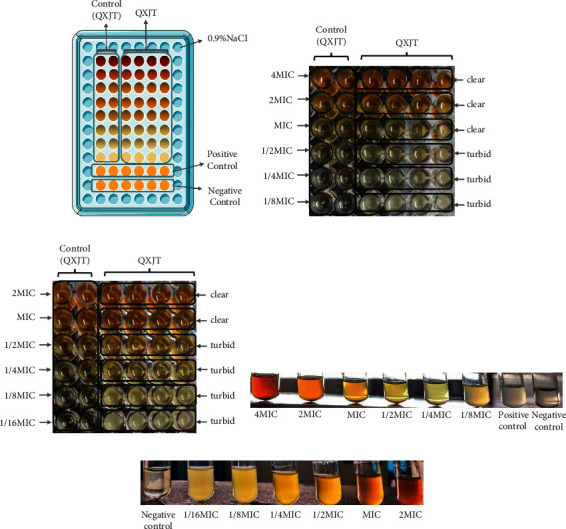
(a) 96-well plate plus sample: control (QXJT) means QXJT was continuously twofold diluted without adding bacteria liquid as control; positive control meant broth is added with bacteria without drugs; negative control meant broth is added without bacteria without drugs. (b) QXJT against MRSA2007118 (broth microdilution method results); MIC = 18.75 mg/ml. (c) QXJT against MRSA2008043 (broth microdilution method results); MIC = 37.5 mg/ml. (d) QXJT against MRSA2007118 (broth macrodilution method results); MIC = 18.75 mg/ml. (e) QXJT against MRSA2008043 (broth macrodilution method results); 37.5 mg/ml.

QXJT had antimicrobial effects on MRSA. According to Figure 4, the liquid in the MIC well was straightforward and without bacterial growth, which was not different from the control (QXJT). The MICs of QXJT against MRSA2007118 and MRSA2008043 were 18.75 mg/ml and 37.5 mg/ml, respectively. QXJT exhibited significant antimicrobial activity against the tested MRSA. Each experiment was independently repeated three times.

### 4.3. A Time-Kill Assay

**Figure 5:**
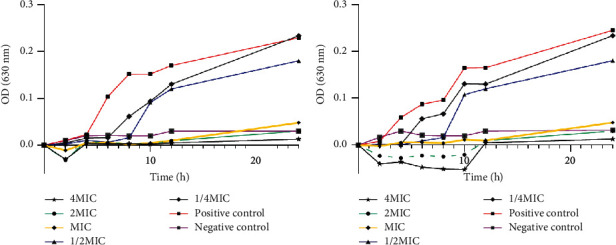
MRSA was incubated at different concentrations of QXJT from 1/4MIC to 4MIC. OD630 values were measured at 0, 2, 4, 8, 10, 12, and 24 h after treatment. Each experiment was repeated twice. (a) A time-kill assay of MRSA2007118. (b) A time-kill assay of MRSA2008043.

To further verify the inhibitory effect of QXJT on MRSA, a time-kill curve was made by detecting the OD values after treatment of MRSA with different concentrations of QXJT. The data were analyzed as shown in Figure 5. The time-killing curves of the 2 MRSA strains were similar. When MRSA was treated with 1/4 MIC, the OD630 nm value did not decrease significantly after 24 hours, but it served to delay the logarithmic growth period of MRSA. When MRSA was treated with 1/2 MIC, the OD630 nm value after 24 h could be significantly reduced. When the bacteria were treated with concentrations of MIC, 2 MIC, and 4 MIC, the OD630 of MRSA was almost unchanged between 0 and 24 h. These data suggested that QXJT could inhibit MRSA growth with a significant concentration dependence.

### 4.4. Checkerboard Assay

The MICs of OX against MRSA2007118 and MRSA2008043 were 4 mg/ml and 1 mg/ml, respectively. The synergistic effect of QXJT in conjugation with OX was evident in the FIC indices shown in Table 3. Overall, test microorganisms showed FIC index (=0.5) on the checkerboard assay, which indicates a synergism effect in the combination of QXJT and OX.

### 4.5. Transmission Electron Microscopy

**Figure 6:**
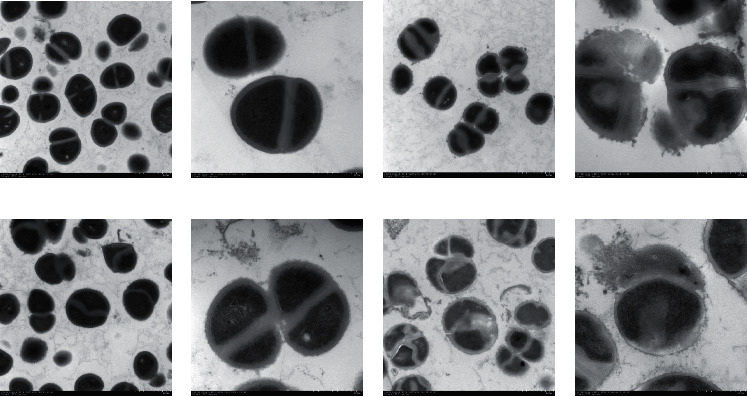
(a, b) Transmission electron micrographs of untreated MRSA cells showing normal cell shapes. (c, d) Transmission electron micrographs of treated with 1/2 MIC of QXJT for 24 h. (e, f) Transmission electron micrographs of treated with 1/2 MIC of OX for 24 h. (g, h) Transmission electron micrographs of treated with 1/2 MIC of QXJT + OX for 24 h.

To further observe the damage of QXJT on bacteria and the synergistic effect of QXJT + OX and explore the possible mechanism of action, we observed MRSA2007118 treated with QXJT, OX, and QXJT + OX at 1/2 MIC by TEM, respectively. Untreated cells are shown in Figures 6(a) and 6(b). Normal MRSA organisms were intact, almost round, with continuous cell walls, well-defined edges, and uniform cytoplasmic distribution. MRSA organisms treated with 1/2 MIC QXJT for 24 h showed deformation of the organism, vacuoles in the cytoplasmic region, slight cytoplasmic consolidation, incomplete cell wall, increased surface projections, severe damage to the organism, and cytoplasmic leakage (Figures 6(c) and 6(d)). After 24 h treatment with 1/2 MIC OX, the cytoplasmic consolidation of MRSA bacteriophage was not obvious. The cytoplasm was more uniform, and the cell wall grossness was seen to be damaged to different degrees (Figures 6(e) and 6(f)). After treatment with 1/2 MIC QXJT + OX for 24 h, it was observed that the MRSA bacteriophage was deformed, the cell wall was thin and discontinuous, the bacteriophage was severely damaged, and the cytoplasm was leaked. This indicated that QXJT + OX produced great damage to the cell wall of MRSA organisms, and this damage was more severe than that caused by either OX or QXJT alone. The synergistic nature of the cell wall damage was observed from the microscopic mechanism of the combination of the two (Figures 6(g) and 6(h)), and surprisingly, we observed that, after being treated by QXJT + OX, MRSA adhered together similar to “rose.” This is a unique form of disruption, and it is speculated that the combination of QXJT + OX may have influenced the division of MRSA organisms.

### 4.6. Quantitative Reverse-Transcription PCR

**Figure 7:**
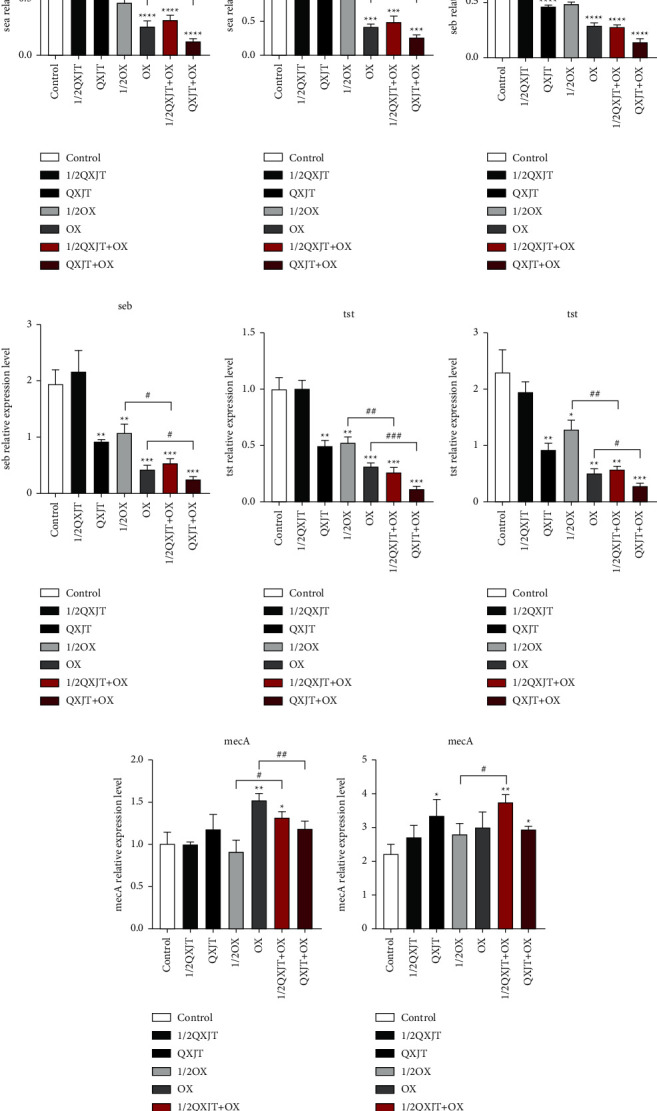
Different concentrations of QXJT, OX, and QXJT + OX were added to the final concentration of 5 × 105 CFU/mL and incubated at 37°C for 24 h. The expression levels of sea, seb, and tst were compared.  ^*∗*^*P* ≤ 0.05,  ^*∗∗*^*P* ≤ 0.01,  ^*∗∗∗*^*P* ≤ 0.001,  ^*∗∗∗∗*^*P* ≤ 0.0001 vs. control group; ^#^*P* ≤ 0.05, ^##^*P* ≤ 0.01, ^###^*P* ≤ 0.001, ^####^*P* ≤ 0.0001 vs. 1/2OX or OX group. (a) Relative gene expression of MRSA2007118 strain sea under different concentrations of drug treatment. (b) Relative gene expression of MRSA2008043 strain sea under different concentrations of drug treatment. (c) Relative gene expression of MRSA2007118 strain seb under different concentrations of drug treatment. (d) Relative gene expression of MRSA2008043 strain seb relative gene expression. (e) MRSA2002118 strain tst relative gene expression under different concentrations of drug treatment. (f) MRSA2008043 strain tst relative gene expression under different concentrations of drug treatment. (g) Relative gene expression of mecA in strain MRSA2007118 under different concentrations of drug treatment. (h) Relative gene expression of mecA in strain MRSA2008043 under different concentrations of drug treatment.

To further understand the interaction between QXJT and MRSA, The gene expression of superantigens was examined. The effect of different concentrations of QXJT, OX, and QXJT + OX on the transcription of sea, seb, tst, and mecA of MRSA2007118 and MRSA2008043 genes was examined by fluorescence quantitative PCR technique, and the results are shown in Figure 7. There was no significant decrease in the transcription of sea, seb, and tst under the addition of 1/2 MIC QXJT, but the transcription of sea, seb, and tst was a statistically significant decrease under the addition of MIC QXJT compared with the control group. This inhibition showed a dose-dependent manner: the higher the dose, the stronger the inhibition. The transcriptions of sea, seb, and tst were also significantly decreased by 1/2 MIC, MIC OX, but the decrease was less than that of 1/2 MIC, MIC QXJT + OX, and the difference was statistically significant.

## 5. Discussion

Natural plants represent an almost unlimited (multitarget) source of active ingredients, and because of the wide variety, we usually rely on clinical or life experiences summarized by the ancients to screen drugs with possible antimicrobial effects for the extraction and study.

QXJT is composed of different kinds and quantities of Chinese medicinal herbs with optimal therapeutic efficacy for a comprehensive regimen, according to the principles of traditional Chinese medicine (TCM). It may have more than one target and disturb bacterial physiology in several different pathways, which is beneficial for inflicting severe damage on the bacterial cell. The phenomenon is similar to polypharmacology [19, 20]. The antibacterial mechanism of QXJT is still unclear. We tried to reveal part of the mechanism by in vitro antibacterial experiment.

Due to the abuse of antibiotics and other factors, bacterial resistance has become more and more serious, which has become a century-old problem that needs to be solved in the future. Because of the high morbidity and mortality associated with MRSA, MRSA infection is considered one of the most challenging infectious diseases, resulting in a worse prognosis of sepsis and higher morbidity and mortality [21, 22]. Takesue et al. [23] demonstrated that the failure rate of vancomycin as first-line drug therapy against life-threatening MRSA disease is increasing. The successive reports of vancomycin heterogeneous resistant SA (hVRSA), vancomycin-mediated resistant SA (VISA), and vancomycin-resistant SA (VRSA) detected worldwide [24] remind us that we may be gradually losing the last line of defense against MRSA infection. The search for novel therapeutic agents that are effective against MRSA is therefore critical to maintaining public health in the future.

In our study, combining MIC and time-kill curve results, we found that QXJT showed significant bacterial inhibition against both clinical isolates of MRSA in a concentration-dependent manner. Active substances such as *Scutellarein*, *Scutellarin, Apigenin*, and *Wogonin 7-O-glucuronide* were detected in the phytochemical analysis that may be associated with the antimicrobial activity of QXJT. Perhaps this is because although the MRSA cell wall has a thick layer of peptidoglycan, this barrier is unable to block these tiny molecular compounds. These compounds on the cell wall of MRSA produce damage [25]. We also found that the MIC of MRSA2007118 to OX was about 4 mg/ml, which is four times higher than that of MRSA2008043, but MRSA2007118 was more sensitive to QXJT than MRSA2008043. MRSA is resistant to penicillin drugs by altering the structure of its cell wall through the mecA resistance gene [26]. But this resistance mutation did not show an advantage in resisting the damage caused by QXJT.

From the results of the checkerboard assay, we conclude that QXJT and OX have a significant synergistic effect (FICI = 0.5). We analyze the results of qPCR which show that QXJT and OX do not inhibit the mecA gene, so the synergistic mechanism of the two is not related to inhibiting the expression of drug resistance genes. By TEM images we observe that QXJT and OX are able to disrupt the cell wall, and QXJT + OX can produce greater damage to the cell wall. Through these results and our speculation that the effects of QXJT + OX combinations against MRSA may arise from two distinct types of mechanisms, the first of these is due to QXJT originally having a certain destructive effect on the bacterial cell wall, and this destructive effect can be subtly synergized with the destruction of the cell wall by OX, but the specific target of action is not clear; the second mechanism may arise from the ability of QXJT to inhibit the *β*-lactamase hydrolysis and restore MRSA sensitivity to OX.

Earlier studies focused more on the anti-inflammatory, antioxidant, and antibacterial effects of natural drugs [27–30]. Until recently, several researchers in diverse domains have expressed interest in strategies that target pathogen virulence factors [31]. It contradicts the antimicrobial effect of traditional antibiotics. Because virulence factors are not always required for bacterial survival, the antiviral method is under less pressure to target bacteria and is less vulnerable to bacterial resistance [13, 32].

Studies have shown that the protective effect of natural drugs such as baicalin against lethal infection with enterohemorrhagic *Escherichia coli* may be through direct interaction with Shiga-like toxin 2 [33]. Baicalin showed a significant inhibitory effect on the pathogenic factor hla of *Staphylococcus aureus* [34]. In our study, we found that QXJT + OX significantly inhibited the virulence factors such as sea, seb, and tst of MRSA in a concentration-dependent manner, and QXJT + OX could inhibit the virulence factors such as sea, seb, and tst more significantly. The exact mechanism remains unclear, but this regulatory role in virulence factors may provide new strategies for exploring new antibiotic drugs.

## 6. Conclusions

This article is based on a famous formula for treating sepsis caused by acute purulent infections of the face, hands, and feet: QXJT (a combination of natural drugs), created by Chen Shigong, a leading TCM surgeon with over 700 years of clinical application. In this study, QXJT showed good antimicrobial activity against a clinical isolate of MRSA. We found a significant synergistic effect of QXJT and OX from the combined form of traditional Chinese medicine and Western medicine, which may be related to the mechanism of action of QXJT to disrupt the cell wall of MRSA. However, its effect on the cell membrane cannot be excluded and still needs further study. We found that QXJT and QXJT in combination with OX could inhibit the expression of MRSA virulence factor but had no significant effect on MRSA resistance gene expression without significant effects, providing a new strategy for exploring new antibiotic drugs.

## Figures and Tables

**Table 1:** Chemical identification.

No.	RT (min)	Name	Formula	Ion	Cal. m/z	Mea. m/z	Error (ppm)	MS/MS
1	1.78	Uridine	C_9_H_12_N_2_O_6_	[M-H]	243.0622	243.062	3.445	243.062, 110.0271
2	4.744	Scutellarin	C_21_H_18_O_12_	[M-H]	461.0725	461.0733	4.007	461.0733, 285.0403
3	5.886	Luteolin-7-O-*β*-D-glucuronide	C_21_H_18_O_12_	[M-H]	461.0725	461.0731	3.574	461.0731, 285.0398
4	6.155	Luteolin	C_15_H_10_O_6_	[M-H]	285.0404	285.0398	1.528	175.0409, 151.0047, 133.0307
5	6.41	Polyphyllin VI	C_39_H_62_O_13_	[M + H]	739.4263	739.4256	-3.091	721.4129, 577.2560
6	6.708	Apigenin 7-O-beta-D-glucuronide	C_22_H_20_O_11_	[M-H]	459.0932	459.0933	2.423	459.0933, 268.0372
7	6.85	Scutellarein	C_15_H_10_O_6_	[M-H]	285.0404	285.0408	5.036	285.0408, 255.0243
8	7.007	Wogonin 7-O-glucuronide	C_22_H_20_O_11_	[M-H]	459.0932	459.0923	0.244	459.0923, 283.0610, 268.0372
9	8.355	Apigenin	C_15_H_10_O_5_	[M-H]	269.0455	269.0461	6.133	225.0551, 151.0555

**Table 2:** Primer sequences for real-time RT-PCR.

Target genes	Primer sequences (5′-3′)	
	Forward primer	Reverse primer
tst	CCGGGATCCCATTTGAATGAAGGAGA	GCCCTCGAGTATTGAGTTAGTGAGGAT
sea	ATGGTGCTTATTATGGTTATC	ATGGTGCTTATTATGGTTATC
seb	TGTTCGGGTATTTGAAGATGG	CGTTTCATAAGGCGAGTTGTT
mecA	GTTGTAGTTGTCGGGTTT	TTTATCGGACGTTCAGTC
16s rRNA	GTTCGCAAGAATGAAACTCA	CCCAACACCTTACGGCAC

**Table 3:** QXJT and OX against MRSA.

MIC strains	QXJT(mg/mL)		OX(mg/mL)	
MRSA2007118	18.75		4	
MRSA2008043	37.5		1	

	CIC	FIC_QXJT_	FIC_OX_	FICI
QXJT + OX	4.69 mg/mLQXJT + 1 mg/mLOX	0.25	0.25	0.5, synergism
	9.38 mg/mLQXJT + 0.25 mg/mLOX	0.25	0.25	0.5, synergism

MIC, minimum inhibitory concentration; CIC, combined inhibitory concentration; FIC, fractional inhibitory concentration; FICI, fractional inhibitory concentration index.

## Data Availability

Article data or supplementary data may be requested via e-mail to the author Siyuan Lv (lvsiyuan2008@126.com) and will be shared with applicants.
